# Early recognition of multiple sclerosis using natural language processing of the electronic health record

**DOI:** 10.1186/s12911-017-0418-4

**Published:** 2017-02-28

**Authors:** Herbert S. Chase, Lindsey R. Mitrani, Gabriel G. Lu, Dominick J. Fulgieri

**Affiliations:** Department of Biomedical Informatics, Columbia University Medical Center, PH-20, 622 West 168th street, New York, NY 10032 USA

**Keywords:** Early Diagnosis [E01.390], Diagnostic errors [E01.354], Diagnosis, Computer-assisted [E01.158], Electronic health records [E05.318.308.940.968.249.500], Natural language processing [L01.224.050.375.580], multiple sclerosis [C10.114.375.500]

## Abstract

**Background:**

Diagnostic accuracy might be improved by algorithms that searched patients’ clinical notes in the electronic health record (EHR) for signs and symptoms of diseases such as multiple sclerosis (MS). The focus this study was to determine if patients with MS could be identified from their clinical notes prior to the initial recognition by their healthcare providers.

**Methods:**

An *MS-enriched cohort* of patients with well-established MS (*n* = 165) and controls (*n* = 545), was generated from the adult outpatient clinic. A *random sample cohort* was generated from randomly selected patients (*n* = 2289) from the same adult outpatient clinic, some of whom had MS (*n* = 16). Patients’ notes were extracted from the data warehouse and signs and symptoms mapped to UMLS terms using MedLEE. Approximately 1000 MS-related terms occurred significantly more frequently in MS patients’ notes than controls’. Synonymous terms were manually clustered into 50 buckets and used as classification features. Patients were classified as MS or not using Naïve Bayes classification.

**Results:**

Classification of patients known to have MS using notes of the *MS-enriched cohort* entered after the initial ICD9[MS] code yielded an ROC AUC, sensitivity, and specificity of 0.90 [0.87-0.93], 0.75[0.66-0.82], and 0.91 [0.87-0.93], respectively. Similar classification accuracy was achieved using the notes from the *random sample cohort*. Classification of patients *not yet known* to have MS using notes of the *MS-enriched cohort* entered *before* the initial ICD9[MS] documentation identified 40% [23–59%] as having MS. Manual review of the EHR of 45 patients of the *random sample cohort* classified as having MS but lacking an ICD9[MS] code identified four who might have unrecognized MS.

**Conclusions:**

Diagnostic accuracy might be improved by mining patients’ clinical notes for signs and symptoms of specific diseases using NLP. Using this approach, we identified patients with MS early in the course of their disease which could potentially shorten the time to diagnosis. This approach could also be applied to other diseases often missed by primary care providers such as cancer. Whether implementing computerized diagnostic support ultimately shortens the time from earliest symptoms to formal recognition of the disease remains to be seen.

## Background

Accurate and timely medical diagnosis is the sine qua non for optimal medical care. Unfortunately, diagnostic error, which includes delayed, incorrect or missed diagnosis, is common and accounts for a significant proportion of medical error [1, 2]. Studies looking at a variety of sources such as malpractice claims, autopsy studies and manual review of patients’ notes have revealed that misdiagnoses of disease as a common problem that leads to harm including death. Frequent errors include failure to order or interpret a test, follow-up on finding an abnormal test result or complete a proper history or physical exam [3, 4]. In the outpatient setting, the overall missed diagnosis rate has been estimated to be 5% [5, 6] and has been reported to be as high as 20% for various cancers [7] and chronic kidney disease [8].

Primary care providers, who are usually first to see patients with medical complaints, are well aware that a root cause of diagnostic error is inadequate knowledge and failure to consider a diagnosis [9–13]. They cannot be expected to have mastered the signs and symptoms of the literally thousands of conditions, many belonging to a rarified domain of specialists. With the sustained exponential growth of medical knowledge, accurate diagnosis of patients’ conditions seems increasingly unachievable without assistance from computerized diagnostic support [14].

There are several diagnostic tools such as Isabel® (Isabel Healthcare Inc., USA), Watson® (IBM, Inc. USA), and DxPlain® (Massachusetts General Hospital, Laboratory of Computer Science, Boston, MS) that return a differential diagnosis based on signs, symptoms and demographic information entered by the provider. Although these tools have been shown to improve diagnosis [15–18] they are underutilized for a variety of reasons including the necessity of actively entering data and lack of integration into the EHR [19, 20].

A diagnostic tool that raises awareness of a specific disease could be integrated into the EHR. Illnesses characterized by abnormalities in structured data, easily accessible to a computer, such as laboratory values or vital signs, could be directly diagnosed by mining the patient’s data. Some examples include identification of patients with chronic kidney disease [21, 22], acute kidney injury [23, 24], anemia [25] and sepsis [26].

Many diseases, however, are not characterized by abnormalities manifested in routine laboratory tests. Consider neurological diseases, such as dementia, multiple sclerosis, myasthenia gravis or Parkinson’s disease, or cancer, such as pancreatic or ovarian, illnesses characterized in their earliest stages by subtle signs and symptoms [27–30]. Implementing a diagnostic assistant to achieve early identification of patients with these illnesses is far more challenging because the computer would have to “read” the unstructured narrative portion of the medical record to identify the signs and symptoms characteristic of the particular disease using natural language processing (NLP). Based on the presence or absence of these signs and symptoms, an algorithm could provide an estimate of the likelihood that the patient had the illness in question.

The goal of this study was to test the hypothesis that patients with diseases characterized by subtle signs and symptoms could be identified early in the course of their illness using NLP of the narrative portion of the clinical notes in the EHR. We chose multiple sclerosis (MS) to explore this hypothesis because a formal diagnosis of MS is commonly missed [31] or delayed [32, 33] placing the patient at risk for irreversible complications. Early diagnosis is the key to treatment success [34, 35].

## Methods

### Study population

The patients whose notes were used for this IRB-approved study attend the Associates in Internal Medicine (AIM) clinic at Columbia University Medical Center (CUMC). The clinic is staffed by 150 medical residents and attending faculty who care for approximately 40,000 patients. Two patient cohorts were established for this study: an *MS-enriched cohort* consisted of patients with well-established MS and randomly selected controls (see Table 1). We used the presence (or absence) one or more ICD9 codes for MS (ICD9[MS]), “340”, as the gold standard for the presence (or absence) of MS. The *MS-enriched cohort* was used to identify predictive attributes, develop a classification model and determine achievability of early recognition. A *Random sample cohort* consisted of randomly selected patients from the AIM clinic, some of whom had MS. This cohort was used to identify patients who might have unrecognized MS. Only those MS and controls patients with clinical notes spanning at least two consecutive years in their record were included in the study.

**Table 1:** Two cohorts used for MS classification study

Cohort	Purpose	Unique Patients	
MS-enriched	Identify predictive attributes, develop a classification model, and determine the achievability of early recognition	MS	165
		Controls	545
Random sample	Identify patients with unrecognized MS	MS	16
		Controls	2273

### Extraction and mapping of MS-related signs and symptoms from clinical notes

Classification models were developed using attributes (features) consisting of the well-known signs and symptoms of MS (terms), extracted from patients’ free-text clinical notes using MedLEE, a natural language processing tool that recognizes terms and maps them to the United Medical Language System (UMLS) concept unique identifiers (CUIs) [36]. The patients’ EHR notes were sourced from the clinical data warehouse of CUMC of the New York Presbyterian (NYP) system.

**Fig. 1:**
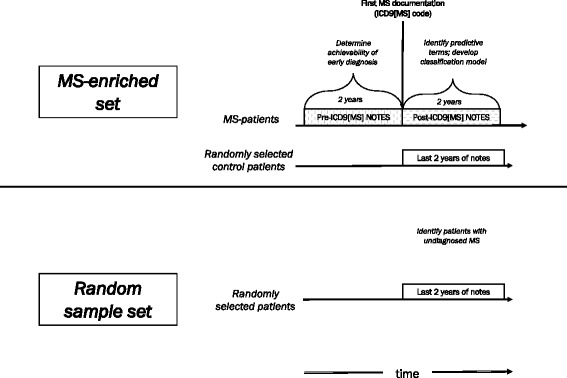
Timing of clinical notes used in classification studies. For MS patients in the MS-enriched set, the notes used were written either before or after the entry of first ICD9 code for MS (IDC9[MS]). For control patients in the *MS-enriched set* and the *Random sample* set, the notes used were those entered within the last two years of the most recent note

We gathered two collections (sets) of notes from the *MS-enriched cohort* based on the timing of the notes in relation to the date of the first entry of an ICD9[MS], (see Fig. 1). The *post-ICD9[MS] set* were notes written about the patients with MS two or more years *after* the initial entry of an ICD9 code for MS (ICD9[MS]) into the EHR, when the diagnosis was clearly established. The *pre-ICD9[MS] set* were notes on these same patients written *prior* to the initial entry of an ICD9[MS] code. Notes of the control patients were entered up to two years prior to the most recent note in the EHR. The *Random sample set* were notes entered up to two years before the last entry in the EHR in patients of the *Random Sample cohort* (see Fig. 1).

### Classification

We trained a classifier to distinguish patients with known MS from those without MS, using the presence or absence of MS-related terms in patients’ notes. We used the Naïve Bayes classification algorithm from the Weka suite to distinguish patients with MS from those without, having previously determined that this algorithm yielded the best results [10]. We assessed classification accuracy by the receiver operating characteristics area under the curve (ROC AUC), sensitivity and specificity and 95% confidence intervals.

## Results

### Characteristics of MS patients and control cohorts

The average age of MS patients in the *MS-enriched cohort* was 47.3 (SD 15.0), significantly lower than controls 54.1 (SD 16.6) (*p* < 0.001). Of the MS patients, 85.1% were female compared to 68.7% of the controls (*p* < 0.001). Both age and gender were subsequently used as attributes in classification.

### Attribute (feature) selection

The first step in building an MS classifier was to identify terms in the patients’ notes that communicated MS (such as “paresis” or “numbness”) that should be more prevalent in patients with MS compared to controls. From the *post-ICD9[MS] set* of notes of the *MS-enriched cohort* unique terms were extracted of which 1,057 were significantly more frequent in MS patients than in controls (Chi-squared analysis and an odds ratio at 5% significance level). Examples of terms with high odds ratios (CI) relating to MS symptoms include weakness in legs, 38.8 (2.3, 693.4), orbital pain 34.9 (2.0, 614.3) and paresthesia foot 38.8 (2.3, 693.4).

To amplify the signal and the potential value of pooling synonymous UMLS terms (CUIs) we manually aggregated individual synonyms into 66 individual buckets. For example, the 14 unique terms representing “paresis” (such as “facial paresis,” “hemiparesis (right),” “hemiparesis (left),” and “limb paresis”) were aggregated into the single bucket “paresis”. The potential attribute strength of each of the buckets was measured by a Chi Squared comparison of the frequency of the terms in MS patients and controls. Of the original 66 buckets, 50 proved to have high attribute strength and were used in the classification modeling (see Table 2). Buckets were grouped to demonstrate the broad range of signs and symptoms of MS as well as the many related but specific categories of loss of function or disability. For example, there are nine different but related buckets of terms that reflect complications and involvement of the motor system.

**Table 2:** Buckets of MS-related UMLS terms used as attributes for MS classification. For each bucket, the frequency of terms in MS patients was compared to controls. The significance of the difference was measured by Chi squared analysis (in parenthesis). Buckets are grouped by common symptoms of loss of function

	BUCKET	SYMPTOM GROUP
1	Bladder dysfunction (115.8)	*Autonomic dysfunction*
2	Constipation (22.4)	
3	Memory (46.4)	*Cognition*
4	Cognitive (19.4)	
5	Ataxia (48.0)	*Coordination*
6	Balance (77.1)	
7	Cerebellar (25.0)	
8	Coordination (89.5)	
9	Dizziness (29.9)	*Dizziness and vertigo*
10	Vertigo (17.2)	
11	Diplopia (43.8)	*Eye and Vision*
12	Nystagmus (48.6)	
13	Optic neuritis (79.2)	
14	Orbital pain (30.0)	
15	Vision (88.9)	
16	Fatigue (30.7)	*Fatigue*
17	Weak (189.0)	
18	Headache (65.8)	*Headache*
19	Migraine (22.5)	
20	Depression (24.2)	*Mood*
21	Mood (36.5)	
22	Pain-musculoskeletal (12.1)	*Pain*
23	Pain-other (7.3)	
24	Atrophy (46.5)	*Motor*
25	Contracture (19.5)	
26	Dysphagia (23.4)	
27	Motor (59.6)	
28	Paresis (87.5)	
29	Reflex (50.6)	
30	Spastic (76.9)	
31	Speech (126.7)	
32	Stiffness (35.6)	
33	Burning (20.0)	*Sensory*
34	Lhermitte’s sign (11.0)	
35	Neuritis (82.8)	
36	Numbness (93.2)	
37	Paresthesia (59.6)	
38	Sensory (67.9)	
39	Tingling (94.5)	
40	Epilepsy (13.3)	*Tremor*, *seizure*
41	Palsy (13.8)	
42	Seizures (33.9)	
43	Tremor (12.9)	
44	Fall (57.3)	*Walking (gait)*
45	Gait (79.2)	
46	Walk (45.8)	
47	Hearing loss (9.0)	*Miscellaneous*
48	Neurologic (43.4)	
49	Neuropathy (22.1)	
50	Sleep (12.6)	

For classification, if a patient had a single mention of any one of a particular bucket terms in any of their clinical notes they were scored a 1 for that bucket (and a 0 if not). Thus, for each of the MS and control patients, attribute columns were filled according to the presence or absence of any one of the several terms within a bucket. If a patient had mention of a term on multiple dates or multiple terms in a bucket, he or she would still receive only a 1 for the bucket.

### Classification of patients known to have MS

The first step to developing the diagnostic assistant was to determine if a classification approach using the extracted signs and symptoms of MS could be used to identify the MS patients already known to have the disease. We developed an MS classification model using the notes of patients with well-established MS (*post-ICD9[MS]* notes of the *MS-enriched set*) and controls. The classification model yielded an ROC AUC, sensitivity and specificity of 0.90 [0.87–0.93], 75% [66–82%], and 91% [87–93%] respectively.

Classification of the *Random sample set* enabled us to obtain a more accurate measurement of sensitivity and specificity of classification of patients known to have MS as well as the true prevalence of MS in our adult population which would allow calculation of positive and negative predictive values [37, 38]. Classification of the *Random sample set*, using the model described above, yielded an ROC AUC, sensitivity and specificity of 0.94 [0.93 - 0.95], 81% [54–95%], and 87% [86–89%], respectively. Based on the observed prevalence of MS in this AIM population of 0.8% (Table 1), and the sensitivity and specificity, the positive and negative predictive values were 3.8% (2.7–7.8%) and 0.15% (0.05–0.47%), respectively.

### Classification of patients *not* known to have MS

We used the classification model, described above, to identify patients of the random sample who might have unrecognized MS. These patients would be amongst those classified as having MS but lacking an ICD9[MS] code. Classification identified 295 such patients, out of a total cohort of 2289. We manually reviewed the notes of a random sample of 45 of these patients and identified four who could have unrecognized MS. For example, one 74-year-old woman complained of migraines, occipital neuralgia, had an abnormal EMG, hand numbness and bowel incontinence, all of which are important symptoms of MS. A second patient, a 75-year old woman complained of peripheral neuropathy, urinary incontinence and visual changes. The remainder of the reviewed patients had one or more medical conditions characterized by the signs and symptoms listed in Table 2, such as cerebrovascular accident, diabetic neuropathy, chronic migraine headaches, seizure disorders, brain cancer, metastatic cancer to the brain and traumatic brain injury. None of the four patients identified above as potentially having MS had any of these neurological conditions. However, inasmuch as none of the four patients suspected to have MS had been seen by a neurologist and were lacking additional follow-up, it was not possible to ascertain whether or not these patients actually had MS.

We next focused on the cohort of patients who were known to have MS (ICD9[MS]) and asked if the classifier would identify these patients as having MS using the notes entered *before* the initial entry of the ICD9[MS] code. Of the 165 patients with MS in the *MS-enriched cohort*, 30 had notes in the EHR up to two years *prior* to the first entry of an ICD9[MS] code. We trained a classifier using the *pre-ICD9[MS] set* of these 30 patients of the *MS-enriched cohort* (Fig. 1) and found that 40% of the patients, with documented MS, were identified by classification using notes entered before the initial ICD9[MS]. The ROC AUC, sensitivity and specificity were 0.71 [0.66 – 0.76], 40% [23–59%] and 97% [93–98%], respectively.

We sought to determine the temporal relationship between the dates of the first ICD9[MS] entry in the *pre-ICD9[MS] set* of the *MS-enriched cohort* and the first recognition of MS by a provider. We manually reviewed the notes of these patients from the time the initial ICD9 code was logged and back in time up to two years before. The dates of the first mention of the illness by the provider in a note and the dates of entry of an ICD9[MS] code were the same in 75% (58–93%) of these patients. Of the remaining patients, the dates of mention of MS (or the possibility of a demyelinating disease) in the notes was on average two months prior to the entry of the first ICD9[MS] code.

## Discussion

### Using NLP to identify patients with MS early in the course of the disease

The purpose of our study was to explore the possibility that patients with MS could be identified earlier in the course of their illness using classification of the signs and symptoms in the clinical notes. Our results demonstrate that earlier identification is feasible. First, we found that 40% (23–59%) of the MS patients in the *MS-enriched set*, with clinical notes entered into the EHR up to two years prior to entry of the first ICD9[MS] code, were classified as having MS. The concordance between the date of entry of an ICD9[MS] code and first reporting of the illness by the provider in 75% (58–93%) of the reviewed patients suggests that classification identified patients who have not yet been recognized as having MS, not simply missing a timely entry of an ICD9[MS] code. Second, manual review of the patients in the *Random sample set*, those classified as has having MS but lacking an ICD9[MS] code identified four of 45 patients’ whose clinical records suggested that they could have MS given that they had signs and symptoms of MS and did not have an obvious neurological condition that mimicked MS. These observations suggest the feasibility of building a diagnostic assistant to identify patients with MS early in the course of their disease perhaps before recognition by the primary provider.

Early diagnosis of a chronic illness through extraction and analysis of clinical terms has been explored previously in studies of celiac disease, well-known to elude diagnosis for years [39]. Two investigations achieved excellent results using different methods to classify patients with celiac disease based on the signs and symptoms present in the medical record [40, 41]. Neither study, however, determined if classification could identify patients before the definitive diagnosis of the disease. A recent study analyzed internet searching histories of patients with pancreatic cancer to identify typical searches and search terms *before* they knew that they had the illness [42]. They did not determine the sensitivity and specificity of using those search terms in identifying patients with pancreatic cancer.

Several groups have sought to identify patients with MS using the data in the EHR, most notably the eMERGE initiatives, seeking to identify patients with various conditions for genetic studies [43]. The eMERGE approach to identify patients with MS used billing codes as well as drugs used to treat MS and entry of “multiple sclerosis” in the notes (using NLP). Davis and coworkers used ICD-9 codes, text keywords, and medications to identify patients with MS and achieved excellent results [44]. Because our long-term goal was to identify patients who might have MS but had not yet been formally recognized as having the illness we specifically avoided using ICD9[MS] codes, drugs that treat MS, or the mention of MS in the notes as attributes for the classifier. Using only signs and symptoms of MS and none of the above-mentioned MS-specific attributes used in these other studies, our classifier achieved a comparable level of accuracy in identifying patients already known to have MS.

### Opportunity for EHR-based clinical decision support

Our results show that perhaps as many as 40% of MS patients could have been identified as having the illness from the signs and symptoms in their notes, well in advance of the initial recognition and assigning of an ICD9[MS] code. The prevalence of MS is sufficiently low, 0.8% (or 1/125 patients) that a busy primary care provider, who first see patients with MS [32], might not consider the illness. Integrating a classification-based clinical decision support tool has the potential to improve recognition of MS by providing a prompt to the provider early in the course of the clinical encounter [45–47].

Any MS diagnostic support tool embedded the EHR, however, would have to clearly communicate that the machine was providing a suggestion, not making a diagnosis. Given our observation that, at best, 4/45 randomly selected patients who were classified as MS but lacked an ICD9[MS] could have MS, the post-test (post-classification) probability would still only be in the neighborhood of 10%. Thus, 90% of the “flagged patients,” those classified as MS, would have some other neurologic condition. Strategies to improve the post-test probability could include removing patients with diseases known to have signs and symptoms similar to MS, such as cerebrovascular accident (CVA), diabetic neuropathy, or migraines. While removing these patients would increase specificity, it would also reduce the sensitivity of classification lessening its utility as a diagnostic prompt given that CVAs and migraines are common in patients with MS [48, 49]. Thus, a post-test probability of 10% may be the best that can be achieved using our approach. That said, a probability of 10%, more than 10 times higher than the prevalence of the disease in the general population, justifies the prompt “consider MS.”

### Limitations of the study

There are several important limitations of our study. First, although there were a sufficient number of patients with MS and notes after entry of the ICD9[MS] code to train a classifier to identify patients with recognized MS, there was an insufficient number who had notes entered *before* the ICD9[MS] code to ascertain if classification could identify them accurately before being recognized as having the disease. Nor could we conclusively determine to what extent we could use the date of the initial ICD9[MS] code as the date of recognition of the disease by the provider. Thus, the classification sensitivity based on the pre-ICD9[MS] notes is likely to be lower than the observed 40%.” Clearly, a much larger set, probably derived from data from several institutions, will be necessary to validate our findings. Second, patients in the random sample whom we suspect may have MS, had received no follow-up or referral to a neurologist (at the time of writing) to ascertain whether or not they had MS. We will have to follow these patients over the next several years before knowing for certain. Third, the classifier was built and tested on our local patient population which has a high proportion of Hispanic, largely female elderly patients. Classification accuracy could be different if applied to a largely white, younger population given the known racial differences in the prevalence of MS [50]. Fourth, we used MedLEE to parse the notes. Many institutions have home-grown term-extractors that would be used to replicate the classification approach described in this study. It is not known how well these other NLP systems would perform [51]. Last, the presence or absence of an ICD9[MS] code was used as the gold standard for the presence of MS in both patient sets. It is known that ICD coding for MS is inaccurate meaning that a proportion of patients with MS will lack an ICD9[MS] code while those who do not have MS receive an ICD9[MS] code [52]. Although this coding inaccuracy influences the accuracy of the classification, it is expected that the accuracy of the classification of patients with known MS would be less than the true accuracy. Thus, our estimates of specificity and sensitively of classification of patients with known MS (ICD9[MS]) are likely an underestimate of the true values. We could have used the eMERGE criteria to identify patients with MS for the classification studies more accurately. However, given that our goal was to identify patients not known to have MS by their providers, we chose not to base the gold standard definition on the eMERGE criteria which are derived from patients with known MS.

## Conclusions

Diagnostic error, a common cause of medical error, might be reduced with diagnostic assistants that recognize diseases by mining patients’ clinical notes for signs and symptoms using NLP. We built a classifier, which identified 40% of patients with MS disease before formal documentation of MS by providers suggesting that earlier recognition of the illness is possible. This approach could be applied to other diseases often missed by primary care providers such as cancer [45]. Whether implementing diagnostic assistants based on this classification approach ultimately shortens the time from earliest symptoms to formal recognition of the disease remains to be seen and will be the focus of future studies.

## Abbreviations

AIM: Associates in internal medicine
CUIs: Concept unique identifiers
CUMC: Columbia University Medical Center
EHR: Electronic health record
ICD9[MS]: ICD9 codes for MS
MS: Multiple sclerosis
NLP: Natural language processing
NYP: New York Presbyterian
ROC AUC: Receiver operating characteristics area under the curve
UMLS: United medical language system
